# Food Insecurity and Adolescent Obesity in the United States: A Social Ecological Analysis of Multi-Level Risk Factors and Structural Inequities

**DOI:** 10.3390/ijerph23040458

**Published:** 2026-04-03

**Authors:** Ogochukwu R. Abasilim, Kenechukwu O. S. Nwosu, Opeyemi O. Akintimehin, Ogochukwu J. Ezeigwe, Odinakachukwu O. Dimgba, Meghna Lama, Amarachi H. Njoku, Nnenna C. Okoye, Elizabeth O. Obekpa

**Affiliations:** 1 Department of Epidemiology and Human Genetics, UTHealth Houston School of Public Health, Houston, TX 77030, USA; kenechukwu.o.nwosu@uth.tmc.edu (K.O.S.N.); ogochukwu.j.ezeigwe@uth.tmc.edu (O.J.E.); odinakachukwu.o.dimgba@uth.tmc.edu (O.O.D.);; 2 Institute of Health Administration, J. Mack Robinson College of Business, Georgia State University, Atlanta, GA 30302, USA; oakintimehin1@gsu.edu; 3 McWilliams School of Biomedical Informatics, UTHealth Houston, Houston, TX 77030, USA; amarachi.h.njoku@uth.tmc.edu; 4 Department of Pharmaceuticals, Gwarimpa General Hospital, Abuja 900108, Nigeria; nnenna.okoye1254@gmail.com; 5 Institute of Implementation Science, UTHealth Houston School of Public Health, Houston, TX 77030, USA; elizabeth.o.obekpa@uth.tmc.edu

**Keywords:** food insecurity, adolescent obesity, health disparities, social ecological model, health equity, poverty, physical activity, race/ethnicity, structural racism, weight status

## Abstract

**Highlights:**

**Public health relevance—How does this work relate to a public health issue?**

- Food insecurity and adolescent obesity are co-occurring public health crises, and this study shows how multiple ecological factors jointly shape obesity risk among U.S. adolescents.
- The findings highlight widening inequities—particularly among low-income, Hispanic, and Black adolescents—underscoring the urgent need for targeted prevention strategies.

**Public health significance—Why is this work of significance to public health?**

- Identifying how food insecurity, poverty, race/ethnicity, and physical activity interact to influence obesity provides actionable evidence for designing multi-level interventions.
- The use of recent, nationally representative NSCH data offers timely insights for policymakers and practitioners seeking to address post-pandemic disparities in adolescent health.

**Public health implications—What are the key implications or messages for practitioners, policymakers and/or researchers in public health?**

- Interventions must address both structural barriers (poverty, racial inequities, access to quality foods) and individual behaviors to effectively reduce obesity in food insecure youth.
- Policymakers and practitioners should prioritize multi-level approaches that improve food environments, support physical activity, and reduce socioeconomic disparities.

**Abstract:**

While the association between food insecurity and adolescent obesity is well-established, the mechanisms through which these co-occurring public health crises are linked remain inadequately understood. Using the Social Ecological Model as a theoretical framework, this study examines how individual (physical activity), interpersonal (household food security), community (poverty level, residence), and societal (race/ethnicity) factors interact to influence adolescent weight outcomes. Cross-sectional data from 37,425 adolescents aged 12–17 years in the 2022–2023 National Survey of Children’s Health using weighted multinomial logistic regression with interaction terms were used. Adolescents experiencing nutrition insecurity (adequate quantity but poor-quality food) had 41% higher odds of obesity (adjusted odds ratio (aOR) = 1.41; 95% CI: 1.20–1.65), while those with food insecurity (insufficient quantity) had 48% higher odds (aOR = 1.48; 95% CI: 1.08–2.02) compared to food-secure peers. Significant effect modification emerged across ecological levels: poverty below the 200% federal poverty level (FPL) significantly amplified the food insecurity–obesity relationship (interaction *p* < 0.001), Hispanic and Black adolescents demonstrated 49% and 78% higher obesity odds, respectively, independent of household food and nutrition security status, and physical activity showed protective effects that varied by food security context (interaction *p* = 0.003). These findings underscore the necessity of multi-level interventions addressing structural inequities alongside individual behaviors to combat adolescent obesity in food-insecure populations effectively.

## 1. Introduction

The coexistence of food insecurity and obesity among American adolescents presents a public health paradox that challenges conventional understandings of malnutrition. While 17.9% of U.S. households with children experience food insecurity, which affects over 13 million youth, simultaneous rates of adolescent obesity have reached 17.2%, representing a syndemic burden that threatens long-term health trajectories [1,2]. This paradox is particularly pronounced among adolescents aged 12–17 years, who face a critical policy gap in nutritional support compared to younger children covered by the Special Supplemental Nutrition Program for Women, Infants, and Children (WIC) and older adults served by senior nutrition programs [3,4]. Previous research reported associations between food insecurity and adolescent obesity; however, findings have been inconsistent, with some studies reporting positive associations and others finding no or inverse relationships [5,6,7]. This inconsistency may reflect the failure to adequately account for effect modification across multiple ecological levels.

The relationship between food insecurity and adolescent weight status operates through complex, multilevel mechanisms that cannot be adequately explained by individual behavioral factors alone. The Social Ecological Model (SEM) offers a comprehensive framework for understanding how factors at individual (intrapersonal), interpersonal, organizational, community, and societal (policy) levels interact synergistically to shape health outcomes [8]. Although organizational factors such as school policies and healthcare systems influence adolescent health, this study focuses on the individual, interpersonal, community, and societal levels, based on available National Survey of Children’s Health (NSCH) data. Grounded in ecological systems theory, the SEM recognizes that human development unfolds within multiple, interdependent contexts. Critically, interventions targeting a single level may be undermined or enhanced by factors at other levels, making it essential to examine how these multilevel influences interact and modify one another’s effects. Applied to the food insecurity–obesity relationship, the SEM shifts the evaluative question from simply “does food insecurity increase obesity risk?” to “for whom, under what circumstances, and through what pathways does this relationship operate?”

At the individual level, mental health conditions, including anxiety, depression, and attention deficit/hyperactivity disorder (ADHD), significantly modify the food insecurity–obesity relationship, with adolescents experiencing very low food security and comorbid emotional or behavioral disorders demonstrating two to threefold increased odds of obesity [9]. This relationship is bidirectional, as greater food insecurity severity is associated with higher rates of mental disorders among adolescents [10,11]. In addition to mental health, other individual-level behaviors and characteristics further shape vulnerability. Physical activity represents another potentially critical modifier, although its protective effects may be constrained among food–insecure adolescents who experience hunger, fatigue, and psychological distress, which limit their ability to engage in regular activity [12]. Sex also influences vulnerability, with girls in food-insecure households showing greater increases in sedentary behavior than boys [12]. These individual-level factors operate within environmental contexts, as low-income neighborhoods where food insecurity is concentrated often lack safe recreational spaces and walkable environments [13]. However, whether physical activity can meaningfully mitigate the risk of obesity for food-insecure adolescents, or whether structural constraints diminish its effectiveness, remains unclear.

At the interpersonal level, parental education emerges as a critical factor that shapes both food security status and adolescent obesity risk, with lower educational attainment being linked to higher rates of food insecurity and increased obesity prevalence [6,14]. Among food-insecure families specifically, mothers often use eating- and weight-related parenting practices, such as restrictive feeding and weight-focused comments, that are associated with maladaptive eating behaviors and heightened obesity risk [14]. Lower parental education is also associated with reduced nutrition knowledge and fewer health-promoting behaviors, affecting how parents procure, prepare, and organize food, which limits access to balanced meals and reinforces obesogenic patterns [15,16]. These patterns highlight how lower educational attainment may compound the challenges families face in maintaining healthy dietary practices under conditions of food insecurity.

At the community level, economic constraints are associated with greater reliance among food-insecure households on energy-dense, nutrient-poor processed foods that maximize satiety while minimizing cost, a pattern associated with elevated obesity risk despite inadequate overall nutrition [17]. These effects are compounded by neighborhood environments, where concentrated poverty often means limited access to healthy food retailers and safe spaces for physical activity [18]. Importantly, these constraints do not manifest uniformly across places: families living below the federal poverty level face disproportionately higher barriers, and these disparities differ sharply by geography. Rural households often face limited grocery availability and transportation challenges, whereas low-income urban households heavily rely on market-purchased foods in densely populated environments where healthy options may be scarce or unaffordable [19].

At the societal level, structural inequities manifest in pronounced racial and ethnic disparities in both food insecurity and obesity, with Hispanic, Black, and American Indian/Alaska Native adolescents experiencing disproportionately elevated rates of each outcome [6,20,21,22]. Federal food assistance programs, such as the Supplemental Nutrition Assistance Program (SNAP), serve as policy-level mechanisms intended to reduce food insecurity; however, their effects on adolescent weight outcomes are mixed and context-dependent. The cyclical structure of monthly benefit issuance may contribute to irregular eating patterns [23,24], and household-level benefit allocation may inadequately meet adolescents’ distinct nutritional needs [25,26]. Moreover, program impacts vary substantially by gender and race/ethnicity, with evidence suggesting protective effects for boys but increased weight risk for girls, alongside differential outcomes across racial and ethnic groups [27,28]. Together, these patterns illustrate that policy interventions do not operate uniformly across populations; their effects are shaped by structural inequities that differentially influence adolescents’ capacity to benefit from available resources.

Although food insecurity and obesity are increasingly recognized as sharing common structural roots, most prior studies have examined individual risk factors in isolation rather than investigating how ecological contexts modify these relationships [5,6,7]. The Social Ecological Model was selected as the guiding framework because it explicitly accounts for the dynamic interplay among individual behaviors, interpersonal dynamics, community conditions, and societal structures [8], an approach that is essential for designing effective multi-level interventions. By systematically testing cross-level interactions, this study aims to generate actionable evidence for policymakers and practitioners seeking to develop comprehensive adolescent obesity prevention strategies that address root causes at multiple ecological levels, rather than focusing narrowly on individual behavior change.

Despite this evidence, critical gaps remain in the literature. Few studies have simultaneously tested cross-level moderation using nationally representative data, modeled severity gradients of food insecurity, or examined multiple potential moderators across ecological levels, including physical activity, household economic status (as measured by the federal poverty level), urban–rural residence, and structural racism as reflected in racial and ethnic disparities. In this study, ‘structural inequities’ refers to systematic disparities in the distribution of resources, opportunities, and power across population groups, driven by historical and ongoing policies and institutional practices, including residential segregation, discriminatory food marketing, and unequal access to healthcare and education, that disproportionately disadvantage certain racial, ethnic, and socioeconomic groups [20]. ‘Multi-level risk’ refers to the simultaneous influence of risk factors operating at different ecological levels (individual, interpersonal, community, and societal) that interact to shape health outcomes, consistent with the Social Ecological Model framework [8,29]. This study addresses these gaps by applying the Social Ecological Model to systematically assess how factors across multiple levels modify the association between household food insecurity and weight status among U.S. adolescents aged 12–17 years. Using data from the 2022–2023 National Survey of Children’s Health, this study investigated: (1) whether household food insecurity is associated with increased odds of overweight or obesity, adjusting for individual-level factors (physical activity, mental health, sex), interpersonal-level factors (parental education), community-level factors (federal poverty level, urban-rural residence), and societal-level factors (race/ethnicity); (2) whether the food insecurity–weight relationship is modified by household poverty level (a community-level factor); (3) how this relationship varies across racial and ethnic groups (a societal-level factor reflecting structural inequities); and (4) whether physical activity (an individual-level factor) moderates the association. By testing these cross-level interactions, this study evaluates the hypothesis that adolescent obesity risk in food-insecure populations arises from intersecting influences across ecological levels, rather than from any single factor operating in isolation.

## 2. Materials and Methods

### 2.1. Study Design and Data Source

This cross-sectional study analyzed data from the 2022–2023 National Survey of Children’s Health (NSCH), a nationally representative survey of U.S. children and adolescents aged 0–17 years. The NSCH employs a stratified random sampling design to generate weighted estimates generalizable to the civilian, non-institutionalized population of children living in households with telephone access. One child is randomly selected per household, with parents or caregivers serving as respondents. Comprehensive methodological documentation for the NSCH has been published elsewhere [30]. Because this analysis utilized publicly available, de-identified secondary data, institutional review board approval was not required.

### 2.2. Study Population

The analytic sample comprised adolescents aged 12–17 years (*n* = 37,425). This age group was selected for three primary reasons. First, adolescence represents a critical developmental period during which dietary autonomy increases, rendering youth more susceptible to obesogenic food environments [3]. Second, adolescents are excluded from federal nutrition policy. They have aged out of programs like WIC targeting younger children; however, they are not served by senior nutrition programs, leaving them disproportionately reliant on household food resources and on SNAP benefits allocated at the household level without age-specific provisions [4,25]. Third, obesity established during adolescence strongly predicts adult obesity and its cardiometabolic sequelae, making this developmental period a high-priority target for prevention [31]. No exclusion criteria based on chronic illness or disability status were applied, as the NSCH surveys the civilian, non-institutionalized population broadly. Adolescents with other chronic conditions or physical disabilities were retained in the sample to maximize generalizability. Missing data were explicitly coded as ‘missing’ for all variables to preserve the representativeness of weighted analyses and avoid bias from listwise deletion.

### 2.3. Conceptual Framework

**Figure 1:**
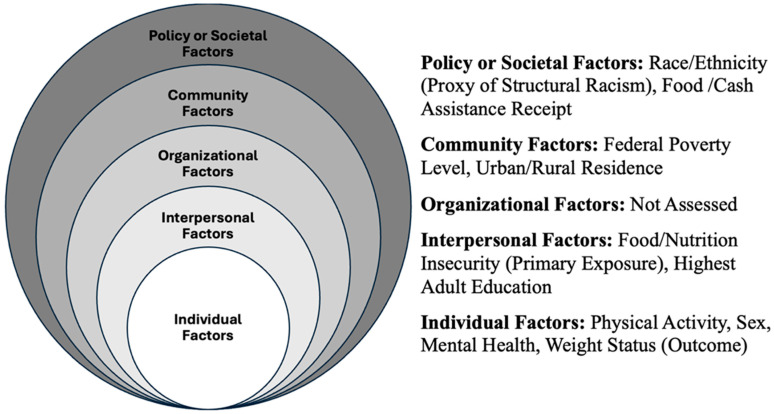
Social ecological model framework.

This study employed the Social Ecological Model (SEM) as its organizing theoretical framework [8]. The SEM posits that health outcomes result from dynamic interactions among individual, interpersonal, organizational, community, and societal factors. Figure 1 maps the study variables onto corresponding SEM levels.

Following the SEM, household food insecurity was conceptualized as an interpersonal-level exposure influencing adolescent weight status (an individual-level outcome) through pathways shaped by factors across various ecological levels. Individual-level factors included physical activity frequency, sex, and mental health conditions. Interpersonal-level factors included household adult educational attainment. Community-level factors comprised the federal poverty level (reflecting local economic constraints) and urban/rural residence (capturing built environment and food access). Societal-level factors included race/ethnicity as a proxy for differential exposure to structural racism [32] and receipt of food or cash assistance programs representing policy-level interventions. Because ecological influences interact rather than operate independently, this study tested cross-level effect modification to determine whether the association between food insecurity and weight status varied by poverty level, race/ethnicity, or physical activity [29].

### 2.4. Measures

#### 2.4.1. Primary Exposure: Household Food and Nutrition Insecurity

Food security status was assessed using the NSCH single-item measure asking: “Which of these statements best describes your household’s ability to afford the food you need during the past 12 months?” Response options were: (1) “We could always afford to eat good nutritious meals” (food secure); (2) “We could always afford enough to eat but not always the kinds of food we should eat” (nutrition insecure); (3) “Sometimes we could not afford enough to eat” (food insecure); and (4) “Often we could not afford enough to eat” (food insecure). Given the small sample size in the “often” category and similar effect estimate across the two insecurity levels, responses 3 and 4 were combined into a single food insecurity category for analysis. This measure captures both food quantity and quality dimensions of food security. While validated multi-item scales such as the USDA Household Food Security Survey Module (HFSSM) provide more comprehensive assessments of food insecurity [2], the NSCH single-item measure captures two critical dimensions: food quantity (‘sometimes/often could not afford enough to eat’) and food quality (‘could always afford enough but not always nutritious food’) [30]. This three-category classification offers a practical advantage by enabling differentiation between nutrition insecurity (adequate quantity, poor quality) and food insecurity (inadequate quantity), a distinction that multi-item binary classifications often obscure. Although this approach may underestimate the prevalence and severity of food insecurity relative to the full HFSSM, it aligns with the NSCH’s design as a broad child health survey and has been used in peer-reviewed analyses of NSCH data [5].

#### 2.4.2. Outcome: Adolescent Weight Status

Weight status was determined using parent/caregiver-reported body mass index (BMI)-for-age percentiles classified as underweight (<5th percentile), normal weight (5th–84th percentile), overweight (85th–94th percentile), or obese (≥95th percentile). Although parent-reported measures may contain errors, previous studies have demonstrated substantial agreement with clinically measured BMI categories, particularly for overweight and obese individuals [33].

#### 2.4.3. Effect Modifiers

Race/ethnicity was categorized as non-Hispanic White, non-Hispanic Black, Hispanic (any race), or other/multiracial. Following Lett et al., [32], race/ethnicity was conceptualized as a social construct that serves as a proxy for differential exposure to structural racism and its downstream health consequences rather than as a biological variable. The federal poverty level (FPL) was categorized into four groups: 0–99% FPL, 100–199% FPL, 200–399% FPL, and ≥400% FPL, representing household income relative to federal poverty thresholds. Physical activity was assessed using the NSCH question: ‘During the past week, on how many days did [child’s name] exercise, play a sport, or participate in physical activity for at least 20 min that made [him/her] sweat and breathe hard?’ This measure captures moderate-to-vigorous physical activity consistent with public health surveillance standards, though it does not assess duration beyond the 20-minute threshold or distinguish between types of activity.

#### 2.4.4. Covariates

Additional variables included in multivariate models were: sex (male/female), highest household adult educational attainment (less than high school, high school/GED, some college/technical school, college degree or higher), residential classification (rural/urban), mental health conditions (parent-reported adolescent diagnosis of anxiety or depression), and receipt of food or cash assistance programs. Receipt of food or cash assistance was categorized based on the total number of program types received (from a list including SNAP, WIC, TANF (Temporary Assistance for Needy Families), free/reduced school meals, and other assistance) as none, 1–2 types, or 3–5 types. This grouping reflects increasing depth of program engagement, with higher counts indicating greater household reliance on the social safety net. These variables were selected based on established associations with both food insecurity and adolescent weight outcomes.

### 2.5. Statistical Analysis

Survey weights from the 2022–2023 NSCH were applied to generate nationally representative estimates. Although this study draws on SEM as a conceptual framework, the statistical approach employed survey-weighted multinomial logistic regression rather than hierarchical (multilevel) modeling. The NSCH sampling design does not provide geographic identifiers (e.g., county or state codes) necessary for nesting individuals within communities or states. Instead, cross-level interactions were used to operationalize the SEM framework by testing whether associations between the interpersonal-level exposure (food insecurity) and the individual-level outcome (weight status) were modified by community-level (poverty, urbanicity) and societal-level (race/ethnicity) factors. Interaction terms were selected a priori based on the SEM framework and existing literature. Each interaction was tested in a separate model to avoid overfitting and multicollinearity. Where interaction strata contained small sample sizes (*n* < 50), estimates are reported but flagged with cautionary notes. Descriptive statistics characterized the distribution of all variables across food security and weight status categories. Pearson’s Chi-square tests assessed associations between categorical variables. Models were adjusted for relevant demographic factors, with the selection of potential confounders informed by existing literature and preliminary univariable analyses. Covariates demonstrating statistical significance (*p* < 0.05) in association with either the exposure or outcome were considered for inclusion in multinomial models. To assess effect modification, separate interaction terms were introduced for poverty level, race/ethnicity, and physical activity in multinomial models. Statistical significance was defined as *p* < 0.05. All analyses were conducted in Stata/SE 18 (StataCorp LLC, College Station, TX, USA).

## 3. Results

### 3.1. Sample Characteristics

A total of 37,425 adolescents aged 12–17 years were included in the analytic sample. Among the participants, 15.21% were overweight, and 14.91% were obese. Most adolescents (65.11%) lived in food-secure households reporting “always enough food”, while 29.08% experienced nutrition insecurity (“always enough food but not always nutritious”), and 5.81% experienced food insecurity (“sometimes” or “often” not enough to eat). The sample consisted of 51.25% males and was racially/ethnically diverse, with 46.27% identifying as non-Hispanic White, followed by Hispanic, non-Hispanic Black, and other/multiracial adolescents. Hispanic adolescents had the highest prevalence of overweight (33.51%) and obesity (36.96%). Most adolescents lived in urban areas (82.61%), and nearly half (48.92%) lived in households where at least one adult held a college degree or higher. Physical activity levels were modest, with only 13.73% reporting daily activity. This group exhibited the lowest prevalence of overweight (13.68%) and obesity (8.69%). The most common activity level was 1–3 days per week (42.37%). Over half of households (52.03%) reported no use of food or financial assistance programs, and 82.43% of adolescents had no reported mental health diagnoses (Table 1).

### 3.2. Association Between Food Insecurity and Weight Status

Household food and nutrition insecurity were significantly associated with adolescent obesity (Table 2). After adjusting for sociodemographic and behavioral variables, adolescents in households with nutrition insecurity (“always enough but not nutritious”) had 41% higher odds of obesity (Adjusted odds ratio (aOR) = 1.41; 95% CI: 1.20–1.65; *p* < 0.001) compared with those in fully food-secure households. Adolescents in households that sometimes could not afford enough food also had an elevated risk of obesity (aOR = 1.48; 95% CI: 1.08–2.02; *p* < 0.05). In contrast, severe food insecurity (“often not enough to eat”) was not significantly associated with obesity (aOR = 0.87; 95% CI: 0.48–1.59). Multiple individual, household, and contextual factors, including poverty level, parental education, physical activity, sex, race/ethnicity, urban/rural residence, and mental health, were independently associated with weight status, highlighting the multilevel determinants of adolescent nutritional outcomes (Table 2).

### 3.3. Effect Modification by Physical Activity

Physical activity significantly modified the association between food insecurity and weight status (*p*-interaction = 0.018; Table 3). Among adolescents experiencing nutrition insecurity, engaging in 4–6 days/week of physical activity was associated with lower odds of underweight (aOR = 0.42; 95% CI: 0.19–0.92; *p* < 0.05). However, in the same group, daily physical activity was associated with an increased odds of being overweight (aOR = 1.92; 95% CI: 1.11–3.32; *p* < 0.05). Among adolescents experiencing moderate food insecurity (“sometimes not enough to eat”), physical activity exhibited a different pattern: those active 4–6 days/week (aOR = 3.78; 95% CI: 1.62–8.84; *p* < 0.01) or daily (aOR = 3.57; 95% CI: 1.40–9.11; *p* < 0.01) had substantially higher odds of overweight. In contrast, among adolescents experiencing severe food insecurity, physical activity appeared protective. Those in the 4–6 days/week category had significantly lower odds of overweight (aOR = 0.05; 95% CI: 0.00–0.48; *p* < 0.05). Adolescents classified as “sometimes not enough food” who were active daily had higher odds of obesity (aOR = 3.07; 95% CI: 1.14–8.28; *p* < 0.05). Overall, the pattern of interaction suggests that physical activity modifies the food insecurity–weight relationship differently across levels of food scarcity, indicating heterogeneity in how adolescents respond to physical activity under conditions of constrained nutrition. In summary, the interaction between food insecurity and physical activity revealed three key patterns. First, among adolescents with nutrition insecurity, physical activity showed no significant protective effect against obesity. Second, among adolescents with moderate food insecurity (“sometimes not enough to eat”), higher physical activity was unexpectedly associated with greater odds of being overweight and obesity. Third, among adolescents with severe food insecurity, physical activity at 4–6 days per week appeared protective against overweight. Reference group: food-secure adolescents with 0 days of physical activity per week; outcome reference category: normal weight. (Table 3).

### 3.4. Effect Modification by Poverty Level

Household federal poverty level significantly modified the food insecurity–weight relationship (*p*-interaction < 0.001; Table 4). Independent of food insecurity, adolescents in the highest-income households (>400% FPL) had substantially lower odds of overweight compared to those in the poorest households (<100% FPL) (aOR = 0.63; 95% CI: 0.48–0.83; *p* < 0.01). The interaction terms demonstrated that nutrition insecurity was most strongly associated with obesity in higher-income households. Among adolescents in households > 400% FPL, nutrition insecurity was associated with more than double the odds of obesity (aOR = 2.14; 95% CI: 1.36–3.38; *p* < 0.01) and elevated odds of overweight (aOR = 2.04; 95% CI: 1.33–3.13; *p* < 0.01). Middle-income adolescents (200–399% FPL) experiencing nutrition insecurity also had increased odds of obesity (aOR = 1.64; 95% CI: 1.08–2.49; *p* < 0.05). In contrast, among adolescents in the poorest households (<100% FPL), nutrition insecurity was not significantly associated with obesity. Adolescents in households at 100–199% FPL who experienced severe food insecurity had markedly higher odds of overweight (aOR = 6.11; 95% CI: 1.26–29.62; *p* < 0.05). Conversely, in the wealthiest households (>400% FPL), adolescents reporting frequent food insecurity had significantly lower odds across weight categories (*p* < 0.001), although these findings should be interpreted cautiously due to small subgroup sizes. In summary, the interaction between food insecurity and poverty level revealed that nutrition insecurity was most strongly associated with obesity among the highest-income households (>400% FPL: aOR = 2.14) and middle-income households (200–399% FPL: aOR = 1.64) while showing no significant association among the poorest households (<100% FPL). Severe food insecurity among adolescents at 100–199% FPL was associated with markedly higher odds of overweight (aOR = 6.11), though this estimate should be interpreted cautiously due to the small subgroup size. Reference group: 0–99% FPL, food-secure; outcome reference category: normal weight. (Table 4).

### 3.5. Effect Modification by Race/Ethnicity

The association between food insecurity and weight outcomes differed significantly by race/ethnicity (*p*-interaction < 0.001; Table 5). Independent of food security status, Hispanic adolescents had significantly higher odds of being overweight (aOR = 1.49; 95% CI: 1.22–1.83; *p* < 0.001) and obese (aOR = 1.54; 95% CI: 1.23–1.95; *p* < 0.001) compared to non-Hispanic White adolescents. Similarly, non-Hispanic Black adolescents demonstrated elevated odds of overweight (aOR = 1.45; 95% CI: 1.12–1.88; *p* < 0.01) and obesity (aOR = 1.78; 95% CI: 1.35–2.35; *p* < 0.01). Hispanic adolescents had significantly lower odds of being underweight (aOR = 0.50; 95% CI: 0.35–0.72; *p* < 0.001). Among non-Hispanic White adolescents (the reference group), nutrition insecurity was associated with significantly higher odds of both being overweight (aOR = 1.33; 95% CI: 1.11–1.60; *p* < 0.01) and obese (aOR = 1.38; 95% CI: 1.15–1.65; *p* < 0.01). Moderate food insecurity (“sometimes not enough food”) exhibited similar patterns, with elevated odds of being overweight (aOR = 1.66; 95% CI: 1.05–2.63; *p* < 0.05) and obese (aOR = 1.70; 95% CI: 1.15–2.52; *p* < 0.01). Hispanic adolescents experiencing nutrition insecurity demonstrated a unique vulnerability to underweight status (aOR = 2.50; 95% CI: 1.29–4.87; *p* < 0.01). Among the non-Hispanic Black adolescents, severe food insecurity (“often not enough food”) was associated with significantly lower odds of obesity (aOR = 0.14; 95% CI: 0.04–0.56; *p* < 0.01), suggesting a protective or inverse relationship in this subgroup. Adolescents classified as “other/multiracial” experiencing nutrition insecurity had elevated odds of obesity (aOR = 1.55; 95% CI: 1.05–2.28; *p* < 0.05), while those reporting severe food insecurity showed significantly lower odds of underweight (*p* < 0.001). In summary, Hispanic and non-Hispanic Black adolescents demonstrated significantly elevated baseline odds of overweight and obesity independent of food security status. Among non-Hispanic White adolescents, both nutrition insecurity and moderate food insecurity were associated with higher odds of overweight and obesity. Hispanic adolescents with nutrition insecurity showed a unique vulnerability to underweight (aOR = 2.50). Among the non-Hispanic Black adolescents, severe food insecurity was associated with significantly lower odds of obesity (aOR = 0.14), a finding that warrants cautious interpretation given the small subgroup sizes. Reference group: non-Hispanic White, food-secure; outcome reference category: normal weight. (Table 5)

## 4. Discussion

This study applied the SEM to examine how individual, interpersonal, community, and societal factors interact to shape the relationship between food insecurity and adolescent weight status. These findings demonstrate that adolescent obesity risk emerges through complex, multilevel pathways that vary substantially across demographic and behavioral contexts. Both nutrition insecurity (adequate quantity, poor quality) and food insecurity (inadequate quantity) were independently associated with elevated odds of obesity, and these associations were significantly modified by poverty level, race/ethnicity, and physical activity.

### 4.1. Individual Level: Complex Physical Activity Interactions

Physical activity demonstrated complex interactions with food insecurity (*p*-interaction = 0.018), which challenged simplistic intervention recommendations. Notably, for nutrition insecurity, physical activity showed no significant moderating effects on obesity, suggesting that poor diet quality is associated with conditions that physical activity alone may not compensate for. This is consistent with evidence that physical activity does not significantly mediate the food insecurity–BMI association [34], and that the protective effects of physical activity against obesity are substantially attenuated in the context of poor diet quality [35]. These findings underscore that individual behaviors operate within (and are constrained by) interpersonal (household food access), community (safe activity spaces), and societal (structural barriers) contexts [36]. Interventions promoting physical activity must simultaneously address nutritional adequacy, rather than treating activity as a standalone

### 4.2. Interpersonal Level: Diet Quality over Quantity

Nutrition insecurity was associated with 41% higher odds of obesity, and moderate food insecurity showed similar effects (aOR = 1.48). In contrast, severe food insecurity was not significantly associated with obesity. This pattern suggests that diet quality, rather than caloric insufficiency, is the central pathway linking food insecurity to obesity. These findings are consistent with recent analyses reporting significant associations between food insecurity and obesity among U.S. adolescents [6,37], and with experimental evidence demonstrating that ultra-processed diets promote excess calorie intake and weight gain independent of other factors [38]. Economic constraints are associated with greater reliance among food-insecure households on energy-dense, nutrient-poor processed foods that maximize calories per dollar [17], a pattern that nonetheless may promote weight gain through insulin resistance, chronic inflammation, and disrupted appetite regulation [39,40]. The substantial effect size for nutrition insecurity underscores that inadequate diet quality, even when quantity appears sufficient, creates metabolic vulnerabilities that manifest as obesity.

Several biological and psychosocial mechanisms have been proposed to explain this association. The chronic stress of food insecurity may activate the hypothalamic–pituitary–adrenal (HPA) axis, promoting cortisol-mediated visceral fat accumulation and stress-induced eating behaviors [41,42]. Adolescents in food-insecure households may develop compensatory overconsumption patterns during periods of food availability following restriction, a “feast-or-famine” cycle [43]. Longitudinal data suggest that household food insecurity during adolescence predicts new onsets of disordered eating and elevated body mass index in young adulthood [44]. These mechanisms may operate simultaneously and could be compounded by the concentration of food deserts and food swamps in low-income neighborhoods, which constrain dietary choices regardless of household-level food security status [18,45,46].

### 4.3. Community Level: The Poverty Paradox

One of the most striking findings was the paradoxical pattern of effect modification by poverty level. Nutritional insecurity showed the strongest association with obesity among the wealthiest families (>400% FPL: aOR = 2.14) and middle-income families (200–399% FPL: aOR = 1.64), while showing no significant association among the poorest households (<100% FPL: aOR = 0.98). This pattern diverges from existing literature predominantly reporting food insecurity–obesity associations among low-income populations [5,28,37,47]. However, emerging evidence suggests that the relationship between diet quality and obesity extends across the income spectrum. Hall et al. [38] demonstrated in a controlled trial that ultra-processed diets promote excess calorie intake and weight gain regardless of economic context, while Juul et al. [48] found that ultra-processed food consumption was associated with excess weight across socioeconomic groups.

Several explanations merit consideration. First, the study measure assessed diet quality (“not always the kinds of food we should eat”) rather than quantity, which among affluent families may capture the consumption of highly palatable, energy-dense ultra-processed convenience foods driven by time constraints, food marketing, and the modern food environment rather than economic deprivation [38,48]. Second, measurement bias may contribute among higher-income families; endorsing this response may reflect greater nutritional awareness rather than genuine food access barriers. Third, the poorest families may benefit from food assistance programs and community resources that buffer against severe dietary inadequacies. Fourth, in neighborhoods with concentrated poverty, household food insecurity compounds broader environmental barriers, creating complex effects differing from episodic dietary quality issues in affluent contexts where food access is abundant [45,46]. Fifth, residual confounding from unmeasured variables, including parental BMI, detailed dietary intake, and genetic predisposition, cannot be excluded. Finally, misclassification of dietary quality may occur if the construct being measured differs systematically across income strata.

This finding, if replicated, has important implications for public health research and practice. Obesity prevention strategies focused exclusively on economically disadvantaged populations may overlook substantial risk among middle- and higher-income adolescents whose dietary quality is compromised by factors other than economic access. Interventions promoting dietary quality and addressing the obesogenic environment of ultra-processed foods are needed across the socioeconomic spectrum. Future research should employ multi-item food security measures alongside detailed dietary assessments to determine whether this pattern replicates, and to identify the specific dietary behaviors underlying this association.

### 4.4. Societal Level: Structural Racism and Policy Limitations

Hispanic and Black adolescents demonstrated elevated baseline obesity risk independent of measured food security, poverty, and other covariates (Hispanic: aOR = 1.58; Black: aOR = 1.49), consistent with exposure to structural racism operating through residential segregation, discriminatory food marketing, and chronic discrimination-related stress [5,20,31,49,50,51]. In this study, race/ethnicity is conceptualized not as a biological risk factor, but as a social construct serving as a proxy for differential exposure to these mutually reinforcing inequitable systems [20,32]. While nutrition insecurity conferred similar relative risks across racial/ethnic groups, the substantially higher baseline obesity risk among Hispanic and Black youth translates to a greater absolute burden in these communities.

The persistence of these disparities despite federal food assistance programs reveals fundamental policy limitations. SNAP shows inconsistent effects across demographic groups and has been linked to a higher consumption of ultra-processed foods among adolescents, suggesting that economic access alone does not guarantee nutritional adequacy when the broader food environment promotes unhealthy options [52,53]. These patterns exemplify how policy interventions that operate within, rather than transform, structurally inequitable systems may fail to address health inequities.

However, using broad racial/ethnic categories as proxy measures has important limitations as it aggregates diverse populations with heterogeneous immigration histories, cultural dietary practices, and experiences of structural racism, obscuring within-group variation that may be critical for understanding disparities. Future research should incorporate direct measures of structural racism, including residential segregation indices, food environment quality metrics, and experiences of discrimination, to move beyond proxy measures [32].

### 4.5. Implications for Public Health

Cross-level interactions indicate that effective interventions must be implemented simultaneously at multiple ecological levels. Individual-level nutrition education and physical activity promotion may offer benefits but cannot replace access to adequate nutrition. Interpersonal-level interventions should support households in making informed food choices through increased SNAP benefits, nutrient-dense food incentives, more frequent distributions, and age-specific allocations. Community-level interventions require investment in food infrastructure and safe recreational spaces across the economic spectrum, not solely in low-income neighborhoods, given the paradoxical findings on poverty. Societal-level interventions must confront structural racism through anti-discrimination policies, equitable resource distribution, and dismantling segregation in housing, education, and employment, as race-neutral policies within structurally inequitable systems perpetuate disparities.

### 4.6. Strengths and Limitations

A key methodological strength is the use of a granular, multi-category measure of household food and nutrition insecurity, which distinguishes between nutrition insecurity (adequate quantity but limited dietary quality) and food insecurity (inadequate quantity). Unlike the binary secure/insecure classifications used in much prior research, this approach enables the detection of subtler patterns that would otherwise be obscured. For example, adolescents in households reporting “always enough food, but not always the kinds of food we should eat” exhibited significantly higher odds of obesity, demonstrating that diet quality deficits alone—well before food scarcity becomes severe—are sufficient to alter weight trajectories. By isolating this middle category, the study captures the lived experiences of families who may not identify as food insecure in traditional terms yet still face substantial barriers to obtaining nutritious foods. These households often fall outside eligibility thresholds for food assistance programs, meaning that their nutritional risks are systematically overlooked. Distinguishing nutrition insecurity from food insecurity, therefore, not only advances measurement precision, but also identifies a critical, policy-relevant subgroup whose needs would remain invisible if food security were treated as a binary construct.

Several limitations warrant consideration. The cross-sectional design precludes causal inference, which is particularly relevant given the potential for reverse causation in physical activity findings. Parent-reported measures introduce potential bias, with research indicating low agreement between parent-proxy and adolescent self-reports of food security [54,55], which may lead to the underestimation of food insecurity prevalence and misclassification of exposure status. Parent-reported BMI data may involve differential misclassification across socioeconomic groups, as parents of higher socioeconomic status may be more prone to underreporting child weight due to social desirability bias. The single-item food security measure, while enabling a granular three-category classification not available with binary measures, lacks the sensitivity and specificity of validated multi-item instruments such as the USDA Household Food Security Survey Module. This measure may underestimate the prevalence of food insecurity, particularly among households that experience intermittent or seasonal food hardship. Furthermore, a single-item measure may capture different phenomena across income levels: among low-income households, it likely reflects genuine economic constraints on food access, whereas among higher-income households, it may capture subjective assessments of dietary quality influenced by nutritional awareness and health consciousness. This differential interpretation may partly explain the paradoxical poverty findings (Section 4.3), and future research should pair single-item screens with multi-item instruments and dietary assessments to disentangle these distinct experiences. The NSCH does not collect data on parental body mass index, a potentially important confounder given the heritability of obesity and shared household environments. The absence of neighborhood-level variables such as food environment characteristics, school nutrition policies, and local food pricing limits the ability to capture important contextual moderators. The inclusion of adolescents with chronic conditions may introduce heterogeneity in the relationship between food insecurity and weight status. Several interaction strata, particularly those involving severe food insecurity combined with specific poverty levels or racial/ethnic groups, contained small sample sizes, resulting in reduced statistical power and wide confidence intervals. These estimates should be interpreted with caution and viewed as hypothesis-generating. Race/ethnicity categorization obscures within-group heterogeneity and may inadequately capture structural racism exposures. Finally, as a U.S.-based study, generalizability to other national contexts with different food systems and social safety nets is limited.

## 5. Conclusions

The association between household food insecurity and adolescent weight status operates through intersecting influences across individual, interpersonal, community, and societal levels. Food and nutrition insecurity, characterized by adequate food quantity but poor dietary quality, was consistently associated with elevated obesity risk, and these associations were further shaped by poverty level, race/ethnicity, and physical activity. The presence of significant cross-level interactions underscores that adolescent obesity in the context of food insecurity cannot be explained by any single factor but reflects the cumulative and interacting effects of economic constraints, structural inequities, and behavioral environments. These findings highlight several implications for public health practice and policy. Efforts to reduce adolescent obesity must extend beyond individual behavior change and prioritize improvements in dietary quality, reinforcement of age-specific nutritional support within food assistance programs, and investments in community food environments and safe activity spaces. Because associations differed by socioeconomic and racial/ethnic group, strategies aimed at promoting health equity must explicitly address structural racism and the broader social and economic systems that shape access to healthy foods and opportunities for physical activity. Future research should employ longitudinal designs to establish causal pathways linking food insecurity and weight status, incorporate direct measures of structural racism and neighborhood disadvantage, and evaluate multilevel interventions that simultaneously address economic hardship, diet quality, and the social conditions that place certain adolescents at heightened risk. Intervening during adolescence is especially critical, as weight trajectories established during this developmental period often persist in adulthood, with long-term implications for population health. These findings carry several implications for public health policy and practice. First, food assistance programs such as SNAP should consider incorporating age-specific nutritional allocations and incentives for purchasing nutrient-dense foods. Community-level investments in healthy food retail and safe recreational spaces should extend beyond low-income neighborhoods, given the unexpected association between nutrition insecurity and obesity across the income spectrum. Strategies aimed at promoting health equity must explicitly address structural racism through anti-discrimination policies and equitable resource distribution. Finally, surveillance systems should adopt multi-item food security measures that capture both the quantity and quality dimensions to better identify at-risk populations.

## Figures and Tables

**Table 1:** Baseline distribution of U.S. adolescents aged 12–17 years (2022–2023) by weight status (*n* = 37,425).

Descriptive Characteristic	*n* (wt. %)	Underweight *n* (wt. %)	Normal Weight *n* (wt. %)	Overweight *n* (wt. %)	Obese *n* (wt. %)	*p*-Value
BMI Category		2139 (6.25)	24,381 (63.64)	5080 (15.21)	4767 (14.91)	0.000
Food and Nutrition Security Status						0.0000
Always enough food	25,818 (65.11)	1551 (64.5)	178,963 (70.26)	3244 (60.29)	2564 (51.11)	
Always enough food but not nutritious	9171 (29.08)	436 (29.09)	5213 (25.23)	1469 (32.79)	1744 (40.23)	
Sometimes not enough food	1272 (5.00)	73 (5.60)	638 (3.73)	205 (6.11)	283 (7.88)	
Often not enough food	208 (0.81)	14 (1.01)	91 (0.78)	33 (0.82)	56 (0.79)	
Sex						0.0000
Male	19,354 (51.25)	1310 (58.2)	11,901 (48.01)	2706 (52.39)	2883 (60.6)	
Female	18,071 (48.75)	829 (41.8)	12,480 (51.99)	2374 (47.61)	1884 (39.4)	
Race/Ethnicity						0.0000
Hispanic	5927 (27.92)	267 (23.66)	3364 (24.37)	987 (33.51)	1058 (36.96)	
White, non-Hispanic	23,767 (46.27)	1358 (49.09)	16,242 (50.64)	3002 (41.42)	2671 (36.28)	
Black, non-Hispanic	2608 (14.11)	137 (13.37)	1393 (12.38)	416 (14.75)	498 (17.67)	
Other/multiracial	5123 (11.71)	377 (13.88)	3382 (12.61)	675 (10.31)	540 (9.09)	
Federal Poverty Level (%)						0.0000
0–99%	4822 (18.27)	69 (20.35)	597 (15.31)	216 (20.31)	238 (23.36)	
100–199%	6142 (20.14)	268 (17.74)	2980 (17.68)	885 (24.37)	1066 (26.97)	
200–399%	10,828 (29.11)	415 (27.28)	4912 (28.85)	1240 (29.86)	1485 (31.50)	
≥400%	15,633 (32.48)	1387 (34.63)	15,892 (38.15)	2739 (25.46)	1978 (18.08)	
Highest Education of Adults in Household						0.0000
Less than high school	1228 (10.39)	69 (14.53)	597 (8.24)	216 (13.23)	238 (12.15)	
High school/GED	5438 (20.14)	268 (16.60)	2980 (17.04)	885 (22.69)	1066 (29.88)	
Some college/technical school	8333 (20.55)	415 (16.00)	4912 (18.09)	1240 (22.34)	1485 (27.05)	
College degree or higher	22,426 (48.92)	1387 (52.87)	15,892 (55.74)	2739 (41.74)	1978 (30.93)	
Physical Activity						0.0000
0 days	4929 (15.12)	349 (20.57)	2665 (12.61)	718 (15.79)	1019 (21.71)	
1–3 days	15,357 (42.37)	923 (41.54)	9660 (41.19)	2172 (42.64)	2228 (46.96)	
4–6 days	11,437 (28.78)	546 (24.61)	8190 (31.30)	1459 (27.89)	1054 (22.64)	
Everyday	5187 (13.73)	292 (13.28)	3649 (14.90)	673 (13.68)	423 (8.69)	
Residence						0.0000
Urban	27,951 (82.61)	1667 (86.36)	18,343 (83.16)	3721 (81.03)	3436 (80.24)	
Rural	6123 (12.08)	277 (8.50)	3782 (11.23)	909 (13.68)	967 (15.55)	
Missing	3351 (5.31)	195 (5.13)	2256 (5.60)	450 (5.29)	364 (4.22)	
Food or Cash Assistance						0.0000
None	22,988 (52.03)	1395 (54.82)	16,140 (58.36)	2823 (45.60)	2161 (34.61)	
1–2 types	10,957 (36.84)	549 (36.74)	6412 (32.65)	1731 (41.43)	1919 (47.93)	
3–5 types	2649 (11.13)	139 (8.44)	1370 (8.99)	411 (12.97)	590 (17.47)	
Anxiety/Depression						0.0000
Yes	8122 (17.57)	437 (15.04)	4966 (16.90)	1160 (18.05)	1368 (22.50)	
No	29,205 (82.43)	1697 (84.96)	19,364 (83.10)	3910 (81.95)	3380 (77.50)	

**Table 2:** Multivariable logistic regression analyses of the association between household food and nutrition insecurity and weight status among U.S. adolescents.

Variable (Reference Group)	Category	Underweight AOR (95% CI)	Overweight AOR (95% CI)	Obese AOR (95% CI)
Food Security (Ref: Always enough food)	Always enough but not nutritious	1.24 (0.95–1.63)	1.17 (1.00–1.37)	1.41 (1.20–1.65) *
	Sometimes not enough food	1.48 (0.90–2.43)	1.30 (0.94–1.80)	1.48 (1.08–2.02) *
	Often not enough food	1.34 (0.48–3.76)	0.88 (0.39–1.97)	0.87 (0.48–1.59)
Sex (Ref: Male)	Female	0.63 (0.51–0.77) ***	0.78 (0.69–0.89) ***	0.51 (0.44–0.58) ***
Residence (Ref: Urban)	Rural	0.69 (0.55–0.88) **	1.25 (1.07–1.46) **	1.46 (1.21–1.77) ***

Note: AOR = Adjusted Odds Ratio; CI = Confidence Interval. * *p* < 0.05, ** *p* < 0.01, *** *p* < 0.001.

**Table 3:** Association between household food insecurity and weight status moderated by physical activity levels.

Variable	Underweight AOR (95% CI)	Overweight AOR (95% CI)	Obese AOR (95% CI)
Food security: Always enough but not nutritious	1.93 (1.01–3.70) *	0.88 (0.58–1.35)	1.34 (0.94–1.91)
Food security: Sometimes not enough food	0.86 (0.32–2.34)	0.73 (0.37–1.44)	0.95 (0.50–1.81)
Food security: Often not enough food	2.74 (0.39–19.07)	2.62 (0.60–11.37)	2.60 (0.93–7.32)
Interaction: Not nutritious × 4–6 days activity	0.42 (0.19–0.92) *	1.30 (0.80–2.10)	1.04 (0.65–1.65)
Interaction: Not nutritious × Daily activity	0.99 (0.35–2.83)	1.92 (1.11–3.32) *	1.40 (0.80–2.44)
Interaction: Sometimes not enough × 4–6 days activity	1.28 (0.37–4.37)	3.78 (1.62–8.84) **	1.85 (0.80–4.25)
Interaction: Sometimes not enough × Daily activity	4.62 (0.93–22.99)	3.57 (1.40–9.11) **	3.07 (1.14–8.28) *

Note: AOR = Adjusted Odds Ratio; CI = Confidence Interval. Outcome reference category: normal weight. Exposure reference category: food-secure (“always afford good nutritious meals”). Physical activity reference category: 0 days per week. Interaction terms represent the combined effect of food insecurity level × physical activity category. Models adjusted for sex, race/ethnicity, parental education, federal poverty level, urban/rural residence, mental health conditions, and food/cash assistance receipt. Estimates based on interaction strata with small sample sizes (*n* < 50) should be interpreted with caution. * *p* < 0.05, ** *p* < 0.01.

**Table 4:** Association between household food insecurity and weight status moderated by household federal poverty level (FPL).

Variable	Underweight AOR (95% CI)	Overweight AOR (95% CI)	Obese AOR (95% CI)
Not nutritious × 200–399% FPL	0.73 (0.32–1.68)	1.32 (0.87–1.99)	1.64 (1.08–2.49) *
Not nutritious × >400% FPL	0.74 (0.31–1.78)	2.04 (1.33–3.13) **	2.14 (1.36–3.38) **
Often not enough × 100–199% FPL	0.69 (0.11–4.29)	6.11 (1.26–29.62) *	1.15 (0.63–7.38)

Note: AOR = Adjusted Odds Ratio; CI = Confidence Interval. Outcome reference category: normal weight. Exposure reference category: food-secure (“always afford good nutritious meals”). Federal poverty level (FPL) reference category: 0–99% FPL. Interaction terms represent the combined effect of food insecurity level × FPL category. Models adjusted for sex, race/ethnicity, parental education, physical activity, urban/rural residence, mental health conditions, and food/cash assistance receipt. Estimates involving severe food insecurity (“often not enough to eat”) at higher income levels are based on very small subgroups and should be interpreted with caution. * *p* < 0.05, ** *p* < 0.01.

**Table 5:** Association between household food insecurity and weight status moderated by race/ethnicity.

Variable	Underweight AOR (95% CI)	Overweight AOR (95% CI)	Obese AOR (95% CI)
Race: Hispanic (Ref: Non-Hispanic White)	0.50 (0.35–0.72) ***	1.49 (1.22–1.83) ***	1.54 (1.23–1.95) ***
Race: Black, non-Hispanic	0.96 (0.65–1.42)	1.45 (1.12–1.88) **	1.78 (1.35–2.35) **
Interaction: Not nutritious × Hispanic	2.50 (1.29–4.87) **	0.84 (0.59–1.19)	1.06 (0.76–1.49)
Interaction: Often not enough × Black	0.30 (0.03–3.27)	0.28 (0.05–1.34)	0.14 (0.04–0.56) **

Note: AOR = Adjusted Odds Ratio; CI = Confidence Interval. Outcome reference category: normal weight. Exposure reference category: food-secure (“always afford good nutritious meals”). Race/ethnicity reference category: non-Hispanic White. Interaction terms represent the combined effect of food insecurity level × race/ethnicity category. Models adjusted for sex, parental education, federal poverty level, physical activity, urban/rural residence, mental health conditions, and food/cash assistance receipt. Estimates involving severe food insecurity (“often not enough to eat”) at higher income levels were based on very small subgroups and should be interpreted with caution ** *p* < 0.01, *** *p* < 0.001.

## Data Availability

The data analyzed in this study are publicly available from the National Survey of Children’s Health (NSCH), administered by the U.S. Census Bureau on behalf of the Health Resources and Services Administration’s Maternal and Child Health Bureau. The datasets can be accessed at: https://www.census.gov/programs-surveys/nsch/data/datasets.html (accessed on 24 February 2024). No new data was created or generated in this study.
